# Biomarker-based machine learning for malignant transformation in oral potentially malignant disorders: a scoping review

**DOI:** 10.1186/s12903-026-08943-x

**Published:** 2026-06-27

**Authors:** Kazem Habibi-Tanha, Julien Ménès, Jordan Gigliotti, Amal Idrissi Janati

**Affiliations:** 1 Faculty of Dental Medicine and Oral Health Sciences, McGill University, 2001 McGill College Avenue, Suite 500, Montreal, QC H3A 1G1 Canada; 2 University of Rochester – Eastman Institute for Oral Health, Rochester, NY 146203 USA; 3 University of Vermont Medical Health Network – Alice Hyde Medical Centre, Malone, NY 12953 USA; 4 McGill University Health Centre – Research Institute, Montreal, QC H3H 2R9 Canada

**Keywords:** OSCC, OPMD, Biomarkers, Machine Learning, Individualized Risk Assessment, Malignant Transformation

## Abstract

Oral squamous cell carcinoma (OSCC) is often preceded by oral potentially malignant disorders (OPMDs). Despite this known association, the transition from an OPMD to OSCC is complex, unpredictable, and non-linear, making early detection and intervention challenging for clinicians. Histopathological grading, the current standard for risk stratification, is not reliably predictive of malignant transformation (MT), and is subject to significant inter- and intra-observer variability. This scoping review evaluates emerging evidence on the integration of artificial intelligence (AI) and machine learning (ML) with molecular and histopathologic biomarkers to enable individualized risk assessment for MT. Ten retrospective studies incorporating AI/ML algorithms were analyzed, utilizing biomarkers ranging from gene expression panels, biochemical and protein-based markers like S100A7 to image-derived histomorphometric features. These models demonstrated promising predictive accuracy, with histology-derived features showing the greatest clinical feasibility. However, variability in methodologies, lack of prospective validation, and inconsistent demographic reporting limit the generalizability of the findings. This review highlights the need for multimodal biomarker integration, prospective clinical trials, and validation across broader populations. Ultimately, AI/ML-enhanced tools hold significant potential to inform personalized surveillance and treatment decisions in OPMD, but their clinical readiness requires further refinement and robust validation.

**Supplementary Information:**

The online version contains supplementary material available at 10.1186/s12903-026-08943-x.

## Introduction

Oral squamous cell carcinoma (OSCC) is often preceded by oral potentially malignant disorders (OPMDs). Despite this known association, the transition from an OPMD to OSCC is complex, unpredictable, and non-linear, making early detection and intervention challenging for clinicians. In Canada, the 5-year and 10-year survival rates for OSCC are approximately 64% and 56%, respectively [1]. The poor outcomes in patients with OSCC are primarily attributed to late-stage diagnosis (i.e., Stages III & IV) [2, 3]. Even among survivors, quality of life is often compromised due to the high morbidity associated with treatment [4]. In contrast, patients diagnosed at early stages (i.e., Stages I & II) have a markedly better prognosis, with survival rates exceeding 80% [5]. OSCC is frequently preceded by a group of lesions collectively referred to as OPMDs, a term recommended by the World Health Organization (WHO) [6]. OPMDs encompass a range of oral mucosal abnormalities and are linked to an elevated risk of malignant transformation (MT). According to the WHO classification of head and neck cancer tumors, MT is characterized by invasion of the cancer cells beyond the basement membrane, affecting the surrounding tissues in an irreversible manner [6]. Although OPMDs are relatively common, only about 6% progress to oral cancer [7]. The risk of MT is highest within the first five years following detection of an OPMD [8]. Among OPMDs, there are certain clinical and pathologic features that portend a higher risk of MT [9]. In clinical practice, these higher risk lesions are excised and/or ablated while lesions deemed to be lower risk are monitored with serial follow-up examinations. Histopathologic evaluation of oral lesions typically classifies biopsied tissue based on the degree of dysplasia, using the WHO three-tier grading system: mild, moderate, and severe dysplasia [6]. Severe dysplasia is associated with the highest risk of progression to OSCC [6]. However, the grading system has notable limitations. Dysplasia is assessed by examining the extent of abnormal basal layer cell proliferation, which is divided into thirds of the epithelial thickness. This somewhat arbitrary division can be subjective, leading to variability between observers and overlap between different grades (intra- and interobserver variability) [10, 11]. Therefore, there is a critical need for the development of more objective and accurate prognostic tools to support clinical decision-making and improve outcomes. As early diagnosis of OSCC is strongly associated with higher survival rates and improved quality of life, identifying reliable biomarkers and developing standardized tools for early detection and monitoring of disease progression are essential.

Several studies have explored the potential of biomarker panels to predict progression and recurrence of head and neck cancers, including OSCC [12–15]. These biomarkers typically fall into three main categories: protein-based, genetic-based, and histopathology-based. Biomarkers such as S100A7 and Invadopodia are closely associated with the progression of OSCC [16, 17]. These proteins contribute to tumor cell survival, invasion, and angiogenesis by activating pathways such as p38/MAPK and EGFR/Src. Additionally, salivary biomarkers (e.g., IL-8, CD44, CYFRA 21 − 1), histopathological features (e.g., the presence and severity of epithelial dysplasia), and genetic alterations (e.g., mutations in TP53, PIK3CA, and CDKN2A) have been implicated in the MT of OPMDs [18–20]. Moreover, image-based histopathological features including nuclear eccentricity, epithelial parameter, and peri-epithelial lymphocyte counts have shown potential as quantitative tissue-based biomarkers to enhance risk stratification [21, 22]. Recent studies highlight the superior performance of multi-marker panels over single-marker approaches for MT in OPMDs [23, 24].

Over the past decade, the integration of machine learning (ML) and artificial intelligence (AI) into medicine and dentistry has progressed significantly, enhancing clinical decision-making, enabling personalized treatment planning, and improving the efficiency of healthcare resource allocation. AI/ML-driven tools can analyze complex datasets, including clinical, imaging, and molecular information, with high speed and accuracy, allowing clinicians to make evidence-based decisions that were previously impractical in routine settings. AI/ML has shown promise in enhancing early detection and prognostication of oral cancer, thereby improving patient outcomes while reducing unnecessary procedures and associated healthcare costs. Given the critical need for early identification of OPMDs that have a high risk of transforming into OSCC, integrating AI/ML with validated biomarkers offers a powerful approach for individualized risk assessment. This integration can help reduce subjectivity associated with conventional tools used in clinical practice, potentially stratifying patients more effectively, prioritizing those requiring immediate intervention, and minimizing overtreatment in low-risk cases. Consequently, AI/ML-enhanced predictive models may not only improve clinical service quality but also contribute to more sustainable and cost-effective healthcare delivery. However, it is still unclear how effectively AI/ML and biomarkers are currently being used together to assess the risk of MT in OPMDs. While several studies have examined AI/ML in oral cancer detection, this review addresses a previously unexplored area in the literature. Existing reviews have primarily focused on AI/ML-assisted diagnosis of oral lesions from clinical or histological images, or on the general landscape of AI/ML applications in dentistry and oncology. In contrast, the present scoping review specifically focuses on the integrated use of AI/ML with molecularly validated biomarkers as predictors of MT in OPMDs, not merely on diagnosis or screening.

## Methods

This scoping review was guided by the updated methodology for conducting scoping reviews from the Joanna Briggs Institute (JBI) and the Preferred Reporting Items for Systematic Reviews and Meta-Analyses Extension for Scoping Reviews (PRISMA-ScR) [25, 26]. A comprehensive search strategy was developed in consultation with expert medical librarians to ensure high sensitivity in identifying relevant studies for our review. Our search strategy included medical subject headings or equivalent controlled vocabulary and keywords, organized according to the JBI scoping review framework. Specifically, the Population was defined as patients diagnosed with an OPMD. The Concept was defined as the application of AI/ML algorithms in combination with biomarkers for the purpose of predicting MT in OPMDs. The Context encompassed human clinical studies of any design (retrospective or prospective) conducted in any geographic location or healthcare setting, using patient cohorts or clinical datasets with documented longitudinal outcomes. We conducted our search in the following databases: PubMed, Scopus, Web of Science, and Cochrane. The search included articles that were published in English between 2000 and February 1, 2025. No additional restrictions were applied. The full search strategy for each database is provided in the Supplementary materials.

The records obtained through our search strategy were imported to Covidence, which permitted the screening to be performed in a blinded manner by two independent reviewers (KHT, JM) [27]. Duplicate articles were automatically removed by Covidence, as well as manually by KHT and JM. The screening process was conducted in two phases: (1) title and abstract screening, and (2) full-text review. In both phases, KHT and JM independently and blindly screened all records. Any discrepancies identified during either phase were documented within the Covidence platform and were resolved through iterative structured discussion between the two primary reviewers (KHT and JM), during which each reviewer was required to provide explicit rationale referencing the eligibility criteria. In cases where consensus could not be reached, a third reviewer (AIJ) was consulted to make the final decision. Inter-reviewer agreement was assessed using Cohen’s kappa, yielding values of 0.49 and 0.44 during the title/abstract and full-text phases, respectively, indicating moderate agreement in both phases, according to the Landis and Koch scale [28]. Primary sources of disagreement during title and abstract screening included uncertainty about whether a study’s ML application was predictive (prognostic) versus purely diagnostic, and ambiguity regarding whether the biomarkers described met our pre-specified definition. During the full-text phase, disagreements arose most frequently around the eligibility of studies that used AI/ML for image analysis of biopsy specimens without a clearly defined MT endpoint, and studies where biomarkers were incorporated descriptively rather than as algorithmic inputs. As this scoping review spanned multiple technical domains, the iterative discussion and third-reviewer arbitration process mitigated the impact of initial disagreements on final inclusion decisions, allowing for complete agreement on inclusion or exclusion decisions in the present review.

The reviewers developed the following inclusion criteria a priori to avoid selection bias: (1) Histopathological confirmation of OPMD status and progression to OSCC, defined as MT, characterized by invasion of epithelial tumor cells beyond the basement membrane in accordance with the WHO classification; (2) AI/ML utilization to assess the risk of progression of any OPMDs to OSCC; (3) Use of AI/ML algorithms trained on prognostic biomarkers, defined as characteristics measured as indicators of pathogenic processes or biological responses, with an established association with oral malignant transformation in prior peer-reviewed clinical or mechanistic literature. For the purposes of this scoping review, we adopted the NIH/FDA BEST (Biomarkers, EndpointS, and other Tools) framework, defining a biomarker as a characteristic that is objectively measured and evaluated as an indicator of normal biological processes, pathogenic processes, or pharmacologic responses to a therapeutic intervention. We categorized biomarkers into four distinct groups: molecular/protein, genetic, digital/computational pathology-derived biomarkers and biochemical biomarkers. These categories were defined by measurement modality rather than by biological origin, such that protein-based markers measured via immunohistochemistry and nucleic acid-level markers measured via sequencing or expression arrays are treated as distinct categories despite their shared genetic basis, consistent with the NIH/FDA BEST framework [29]; (4) Validation of models using human patient cohorts (retrospective or prospective) or clinical datasets with documented longitudinal outcomes; and (5) Studies solely concerned with oral cancer.

Data extraction for included studies was performed independently by two reviewers (KHT and JM) using a standardized data charting form developed within Covidence. To ensure consistency and comprehensiveness, the extraction form was piloted on the first three studies, after which minor adjustments were made to the model performance categories to better capture the heterogeneity of reporting in our selected studies. Any discrepancies identified during the extraction process were resolved through discussion between the two primary reviewers. When consensus could not be reached, a third reviewer (AIJ) was consulted to provide a final decision, ensuring the accuracy and reliability of the synthesized data.

Although often optional for scoping reviews, a formal quality assessment was performed to address the predictive nature of the included studies. We utilized the Quality Assessment of Diagnostic Accuracy Studies (QUADAS-2) tool to systematically assess risk of bias and concerns regarding applicability across four domains: patient selection, index test, reference standard, and flow and timing. This was performed independently by two primary reviewers. Discrepancies were resolved through consensus or consultation with a third reviewer (AIJ).

## Results

### Characteristics of the included studies

**Fig. 1:**
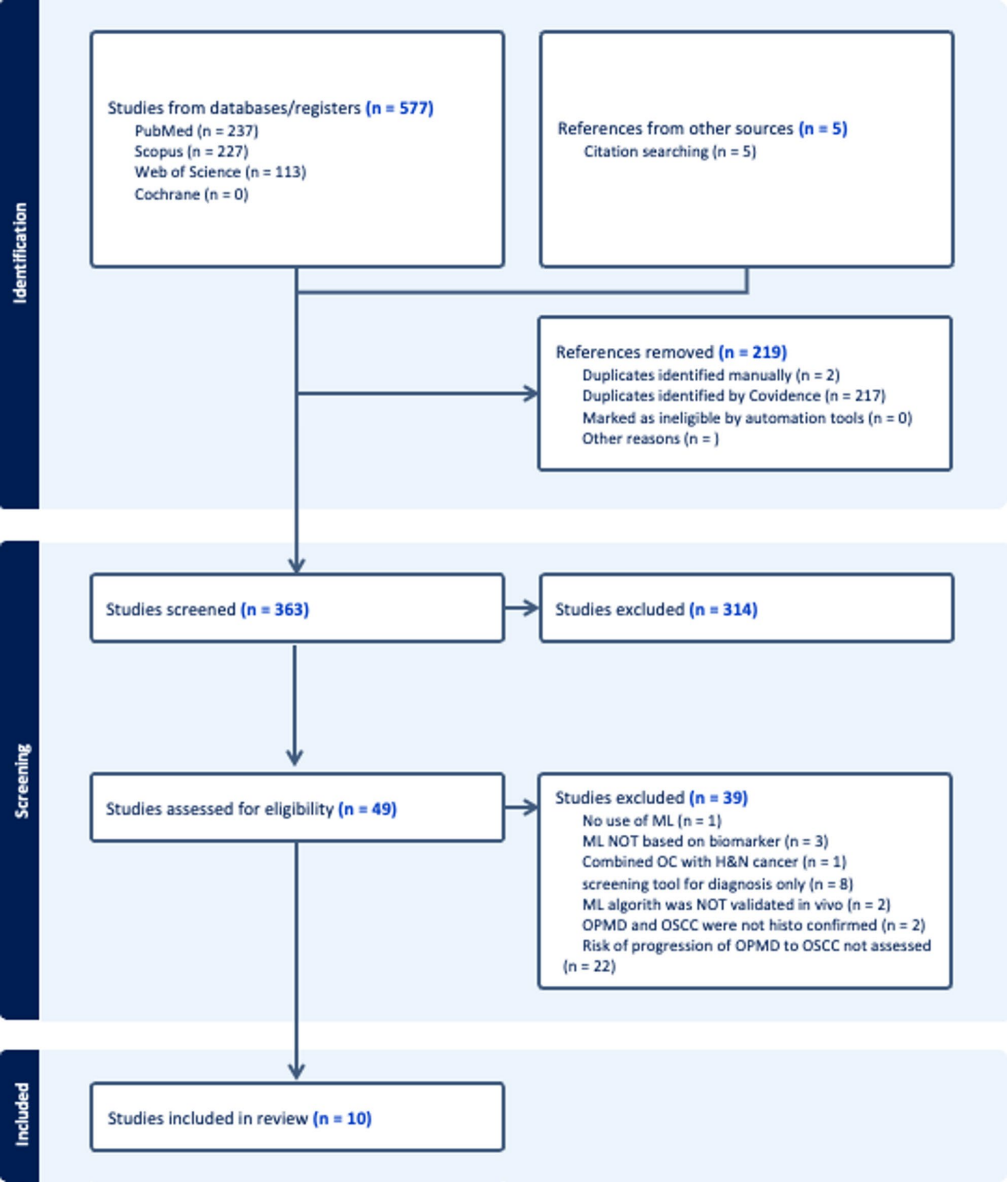
PRISMA Flow Diagram for Selection of Studies Included in the Scoping Review

The search strategy and reference list search retrieved 579 studies. After deduplication, 358 studies entered the first phase of screening. Reviewers agreed on advancing 44 articles to full-text review. The most frequent reasons for exclusion of papers were lack of usage of AI/ML or lack of a combination of biomarkers with AI/ML. Ten studies [22, 23, 30–37] met the eligibility criteria during the final stage of screening and were included in the scoping review (see Fig. 1 for the flow diagram and reasons of exclusion at each stage).

Characteristics of the studies included in this scoping review are presented in Table 1. All studies were retrospective in design and published between 2017 and 2024. Studies used archived biopsy samples to investigate the risk of OPMD progression to OSCC. The first published study, from Hwang et al., was conducted in Toronto, Ontario, Canada, on a total of 150 OPMD cases [30]. Ali et al. used 145 formalin-fixed, paraffin-embedded oral biopsy samples from 95 patients from the Toronto Oral Pathology Service archives at the Faculty of Dentistry, Ontario, Canada [31]. Jing et al. and Shams et al. used multiple publicly available gene-expression datasets to explore biomarker expression [32, 33]. Darling et al. included 48 cases collected from the Northern Ireland Biobank (Queen’s University Belfast, United Kingdom), with further analysis conducted at Mount Sinai Hospital, Toronto, Ontario, Canada [34]. Bashir et al. analyzed 137 OPMD biopsies from the United Kingdom [35]. Mahmood et al. assembled a development cohort of 75 cases and a validation cohort of 121 cases across Sheffield (UK) and UNICAMP (Brazil), totaling 196 cases [21]. Shephard et al. evaluated 193 patients in a development set and 89 in an external validation set in the UK (Sheffield, Birmingham, Belfast), for a total of 282 patients [22]. Ellis et al., representing the smallest cohort of study, included 17 patients with oral epithelial lesions [36]. Lastly, the cohort used by Liu et al. consisted of 68 patients with oral leukoplakia [37]. Overall, the included studies presented sample sizes ranging from 17 to 282 cases, geographically spanning North America, Europe, and South America.

**Table 1:** Characteristics of the Included Studies

First author (Year) Country	Study design & Study period for inclusion	FU period	Number of OPMD cases (slides)	OPMD most frequent sites (%)	Age (in years)	Gender Male %	Type of biospecimens analyzed	Source of biopsy samples	Dysplasia grading system & groups *n* (%)	MT Rate %
Shams (2017) Malaysia [33]	Retrospective bioinformatics and machine learning-based analysis Study period: not specified	-	86	-	-	-	Gene expression profiling (raw microarray data)	University of Texas, accessed online via NCBI GEO	**-**	51/86 = 59.3%
Ellis (2022) UK [36]	Retrospective pilot study Study period: not specified	Progressing group: 2 to 43 months prior to MT Non-progressing group: 43–108 months post biopsy	17: 10 progressing + 7 non-progressing	▪ Lateral tongue (29.4%) ▪ Floor of the mouth (23.5%) ▪ Ventral tongue (23.5%) ▪ Soft palate (11.8%) ▪ Buccal mucosa (5.9%) ▪ Mandibular alveolus (5.9%)	Mean [range]: 59.4 (45–78) years	29.4%	Archival FFPE tissue from incisional biopsies	University of Liverpool/Liverpool University Hospitals NHS Foundation Trust network in the United Kingdom	**WHO 2017**: ▪ Mild: 8 (47.1%) ▪ Moderate: 3 (17.6%) ▪ Severe: 6 (35.3%)	10/17 = 58.8%
Liu (2017) China [37]	Retrospective cohort study Study period; March 2008 to July 2016	Mean in MT group: 23 ± 20 months Mean in non-MT group: 32 ± 31 months	110	▪ Gingiva (40%) ▪ Tongue (25.5%) ▪ Others (34.5%)	Mean [range]: • Training set: 57.68 [26–77] years • Validation set: 58.16 [25–86] years	50.9%	Exfoliative cytology (cells collected via brush biopsy) and tissue from incisional biopsies (FFPE)	Beijing Stomatological Hospital, Capital Medical University, Beijing, China	**-**	7/68 = 10.3%
Hwang (2017) Canada [30]	Retrospective cohort study Study period: not specified	Mean [range]: 44 [0.5–150] months	150	▪ Tongue (66%) ▪ Other sites (34%): unspecified	Mean [range]: 59 [32–88]	50.7%	FFPE biopsy samples & tissue microarrays	Pathology archives from Mount Sinai Hospital, Toronto	**WHO 2017**: ▪ Mild : 65 (43.3%) v Moderate: 54 (36%) ▪ Severe: 31 (20.7%)	60/150 = 40%
Mahmood (2023) Development cohort UK [21]	Retrospective digital pathology-based cohort study Study period: not specified	Min: 5 years	75, excluding HPV-related & verrucous)	▪ Tongue (52%) ▪ Floor of the mouth (20%) ▪ Buccal/labial mucosa (17%)	Median (IQR): 65 (21)	63%		Pathology archives from: ▪ University of Sheffield, UK*	**WHO 2017**: ▪ Mild: 25 (33.3%) ▪ Moderate: 25 (33.3%) ▪ Severe: 25 (33.3%) **Binary Grading**: o Low grade: 33 (44%) o High grade: 42 (56%)	19/75 = 25%
External validation cohort UK & Brazil		Only time to MT reported, median (IQR): 42 (54) months	121	▪ Tongue (60%) ▪ Floor of the mouth (16%) ▪ Buccal mucosa (14%)	Mean (SD): 59 (12.55)	50.5%	4 μm H&E-stained FFPE tissue sections	▪ University of Sheffield, UK (different from*) ▪ Piracicaba Denta School, Brazil ▪ Queen’s University, UK ▪ InHANSE, UK	**WHO 2017**: ▪ Mild: 33 (27.3%) ▪ Moderate: 43 (35.5%) ▪ Severe: 45 (37.2%) **Binary**: o Low grade: 43(36%) o High grade: 78 (64%)	39/121 = 32%
Jing (2022) China [32]	Retrospective bioinformatics and machine learning-based analysis Study period: not specified	-	125	-	-	-	FFPE biopsy-derived RNA expression datasets Cell lines: DOK (OL) and SCC-15 (OSCC)	Public datasets from GEO (GSE26549, GSE85195, GSE85514) TCGA OSCC cohort In vitro: Cell lines from Central Laboratory, Peking University	-	35/125 = 28%
Shephard (2024) UK Internal validation cohort (Training cohort) [22]	Retrospective, multi-center, computational pathology study Study period: 2008 and 2016	Median (IQR): 95 (51) months	202 (279 slides)	▪Tongue (44%) ▪ Floor of the mouth (21%) ▪ Buccal mucosa (13%)	Median (IQR): 64 (19)	54%	4 μm H&E-stained FFPE tissue sections	Archived FFPE slides and clinical records from: ▪ Sheffield (UK)	**WHO 2017**: ▪ Mild: 91 (34%) ▪ Moderate: 104 (39%) ▪ Severe: 75 (28%) **Binary**: o Low grade: 169 (63%) o High grade: 101 (37%)	42/193 = 22%
External validation cohort		Median (IQR): 42 (34) inBirmingham cohort & 47 (34) months in Belfast cohort	89 (89 slides)	▪ Tongue (66%) ▪ Floor of the mouth (13%) ▪ Buccal mucosa (7%)	Median (IQR): 62 (19)	49%		▪ Birmingham (UK) + ▪ Belfast (UK)	**WHO 2017**: ▪ Mild: 30 (34%) ▪ Moderate: 43 (48%) ▪ Severe: 16 (18%) **Binary**: o Low grade: 29 (33%) o High grade: 54(60%)	40/89 = 45%
Darling (2023) Ireland and Canada [34]	Retrospective cohort study Study period: not specified	Mean[range]: 52 [5–14] months	48	▪ Tongue (65%) ▪ Floor of the mouth (27%)	Mean [range]: 61 [25–88]	46%	FFPE biopsy specimens (5 μm sections)	Northern Ireland Biobank (Queen’s University Belfast, UK) Northern Ireland Biobank in the United Kingdom (further analysis of specimens in Toronto, Ontario, Canada)	**WHO 2017**: ▪ Mild: 5 (10.4%) ▪ Moderate: 21 (43.8%) ▪ Severe: 18 (37.5%) **Binary**: o Low grade: 9 (18.8% o High-grade: 35 (72.9%)	30/48 = 63%
Bashir (2023) UK [35]	Retrospective observational study using digital pathology Study period: 2005 to 2016	Mean (SD): 6.51 (5.35) years Min: 5 years	137	▪ Tongue (38.6%) ▪ Floor of the mouth (19.7%) ▪ Buccal mucosa (12.4%)	Mean [range]: 64.6 [25–97] years	61.3%	FFPE H&E-stained slides scanned into WSIs	Archived histological biopsies at the Academic Unit of Oral & Maxillofacial Pathology, University of Sheffield	**WHO 2017 (3-tier)**: ▪ Mild: 41 (29.9%) ▪ Moderate: 53 (38.6%) ▪ Severe: 43 (31.3%) **Binary**: o Low grade: 80 (58.3%) o High grade: 57 (41.6%)	50/137= 37%
Ali (2021) Canada [31]	Retrospective case-control study January 2008 to December 2017	Mean[range]: • Progressing group: 3.5 [1-15.4] years • Non-progressing group: 5.9 [2.9–7.9] years	95 : 49 progressing + 46 non-progressing	Tongue (42%)	Mean [range]: • Progressing group: 65 [29–93] years • Non-progressing group: 56 [27–84] years	Progressing group: 59.2% Non-progressing group: 69.6%	FFPE samples	The Toronto Oral Pathology Service, University of Toronto	**Binary**: o Low-grade: 25 (26.3%) o High grade: 37 (38.9%)	49/95 = 52%

*FU* Follow up, *OPMD* Oral Potentially Malignant Disorders, *OSCC* Oral Squamous Cell Carcinoma, *MT* Malignant Transformation, *FFPE* Formalin-fixed paraffin-embedded, *H&E* Hematoxylin and Eosin, *InHANSE* Institute of Head and Neck Studies and Education, *GEO* Gene Expression Omnibus database, *WHO* World Health Organization, *HPV* Human Papillomavirus, *RNA* Ribonucleic Acid, - Not reported or not applicable

Patient demographics across the included studies were variable, but a slight male predominance was revealed in six of the ten studies [21, 22, 30, 31, 34, 37], with two studies not reporting sex or gender statistics [32, 33]. Mahmood et al. provided sex distributions separately for the development (63% male, 37% female) and validation (59.5% male, 40.5% female) cohorts. Similarly, Liu et al. reported demographic information separately for training and validation sets with 67.9% male for the training set and 45.1% male in the validation set [37]. Ali et al. likewise stratified sex by progression status, reporting approximately 55% males in the progressing group and 63% in the non-progressing group. Shephard et al. reported a gender distribution of 54% males in the internal validation cohort, and 49% males in the external validation cohort. The Ellis et al. study consisted of 12 (70.4%) females and 5 males (29.4%) [36]. Age data were inconsistently reported across studies. Mahmood et al. noted mean ages ranging from 59 to 65 years. Shephard et al. reported median age and interquartile range (IQR) for the internal and external validation cohorts of 64 (IQR: 21) and 62 (IQR: 21) years, respectively. Liu et al. reported the mean age of participants in the training set to be 57.68 ± 13.51 years and the mean age in the validation set to be 58.16 ± 11.48 years. Bashir et al., Ali et al., and Ellis et al. mentioned age distributions without precise summary statistics, and the remaining studies did not specify participant ages. Other clinical covariates, such as smoking history and ethnicity, were sporadically reported. Two studies specified social characteristics of the participants [36, 37]. Ellis et al. reported there were more smokers in the transforming group [36]. Liu et al. reported precise social covariates of their cohorts, such as 57.1% being smokers and 32.1% consuming alcohol in the training set, versus 35.4% and 23.2% in the validation set respectively [37]. Other studies did not systematically report smoking prevalence, cultural practices that may increase risk of OPMD and subsequent OSCC progression, or ethnic composition.

In all studies, lesions were separated into progressing versus non-progressing groups based on whether MT occurred during the follow-up period. Progression rates varied across cohorts, from 10 of 17 cases (58.8%) in Ellis et al., to 50 of 137 cases (36.5%) in Bashir et al. Histopathologic grading was handled in two ways: seven studies [21, 22, 30, 31, 34, 36, 37] used the WHO three-tier dysplasia system (mild, moderate, severe), and one [35] applied a binary low- versus high-risk stratification. Two studies used the same, unstratified, publicly available OPMD dataset [32, 33]. When reported, severe dysplasia accounted for roughly 30–40% of lesions. Regarding the anatomical distribution of lesions, the tongue emerged as the most common lesion site, followed by floor of mouth and buccal mucosa. Mean follow-up was reported in seven of ten studies: Hwang et al. averaged 44 months, Bashir et al. 6.51 years (± 5.35 SD), Shephard et al. reported a median and interquartile range (IQR) follow-up period in months of 95 (IQR follow-up: 51) for the internal validation cohort, and for the two-center external validation cohort of 42 and 47 (IQR follow-up: 35) and Darling et al. 52 months, while Ali et al. reported stratified mean follow-up durations of 3.5 years for progressing and 5.9 years for non-progressing cases. Two studies did not report average follow-up at all, but Mahmood et al. reported a minimum follow-up period for their development cohort (Table 1).

### Quality assessment of individual studies

**Fig. 2:**
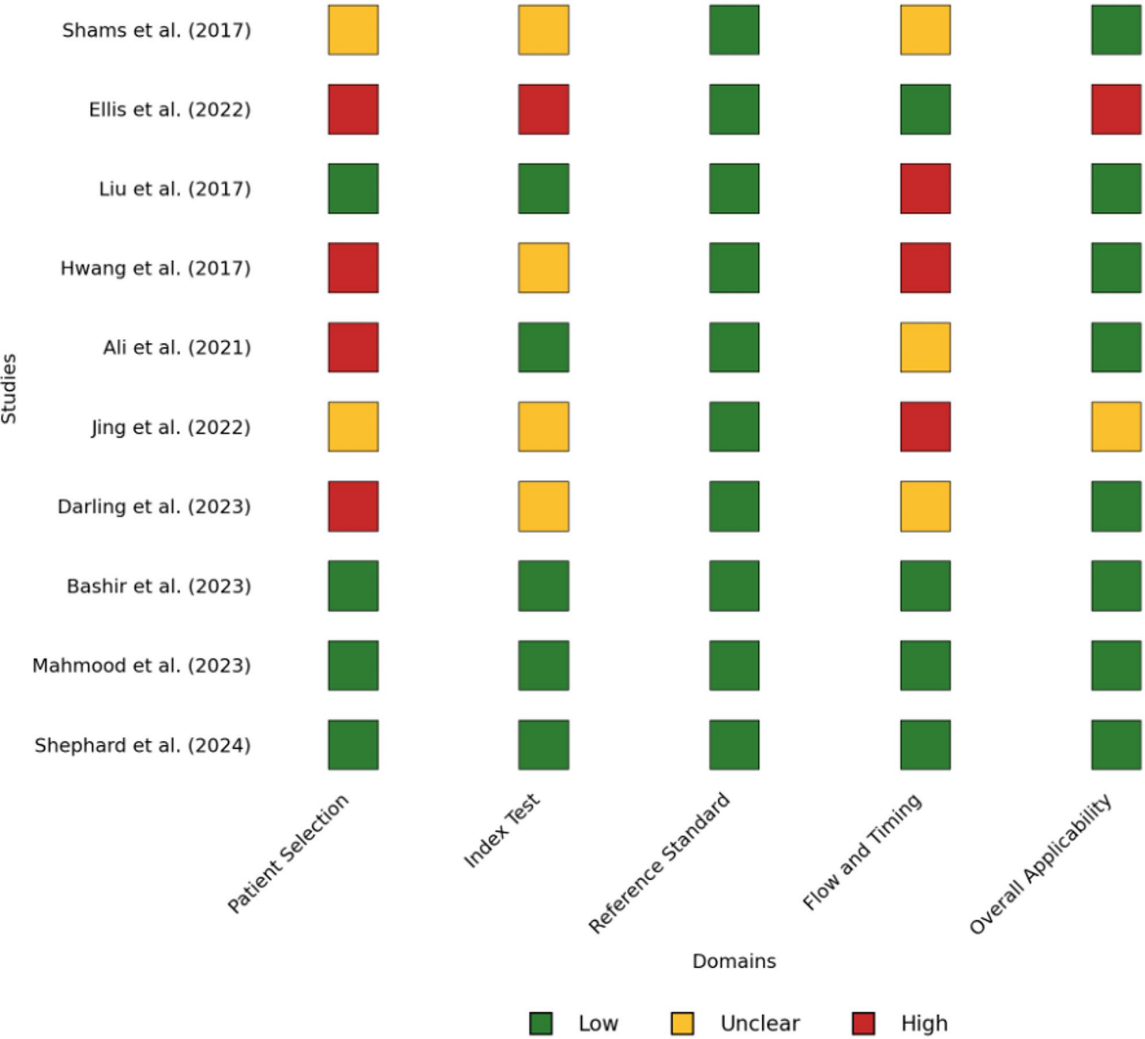
Risk of Bias and Applicability Concerns Summary for Included Studies Using QUADAS-2 Tool

The methodological quality of the ten included studies was assessed using the QUADAS-2 tool (Fig. 2). Across the four QUADAS-2 domains, the Reference Standard domain showed the lowest overall risk of bias, with 10 of 10 studies (100%) rated as low risk, because all studies utilized histopathological confirmation of MT with defined criteria. In the Patient Selection domain, 4 of 10 studies (40%) were rated as high risk, 2 of 10 (20%) as unclear risk, and 4 of 10 (40%) as low risk. The high-risk ratings were driven predominantly by retrospective case-control designs, which may not reflect real-world clinical prevalence and can introduce spectrum bias, potentially inflating apparent model performance [30, 31, 34, 36]. In the Index Test domain, 4 of 10 studies (40%) were rated as unclear risk, largely due to insufficient reporting of pre-specified thresholds, blinding of the index test to outcome status, or inadequate description of the biomarker measurement process. In the Flow and Timing domain, 6 of 10 studies (60%) were rated as high or unclear risk due to inconsistent follow-up durations, raising concerns that some lesions classified as “non-progressing” may simply have had insufficient follow-up to manifest transformation. These domain-specific biases have direct implications for reported model performance. High patient selection bias is known to artificially inflate AUROC and sensitivity estimates, as the case-control enrichment of progressor and non-progressor cases does not reflect the low base rate of MT observed in clinical populations [38]. Accordingly, the performance metrics reported in these studies should be interpreted with caution and not extrapolated to clinical prediction without prospective recalibration.

### Identified biomarker categories

As shown in Table 2, studies employed various biomarkers within four main biomarker categories: morphological biomarkers, protein immunohistochemistry (IHC) markers, gene expression panels and biochemical biomarkers. Mahmood et al., Bashir et al., and Shephard et al. derived quantitative morphological metrics from histologic or confocal images to stratify OPMD risk. The protein IHC group included Hwang et al., Darling et al., and Ali et al. Hwang et al. and Darling et al. both utilized S100A7 (Psoriasin), an inflammatory protein marker, while Ali et al. investigated Cortactin (actin-regulatory protein), Tks5 (tyrosine kinase substrate), and MMP14 (matrix metalloproteinase involved in inflammation). Additionally, Jing et al. and Shams et al. used gene expression panels to identify seven genes involved in MT of OPMDs [32, 33]. Liu et al. employed DNA index which measures the DNA content of cells through DNA-image cytometry to identify aneuploidy [37]. Lastly, Ellis et al. developed an ML pipeline-based Fourier-transform infrared (FTIR) spectroscopy [36]. FTIR utilized infrared light over a broad spectrum to assess the chemical profile of epithelial tissues. Different molecules vibrate at varying frequencies depending on their chemical composition. When infrared light is applied, molecules absorb radiation at wavelengths that correspond to their specific vibration frequencies.

**Table 2:** Overview of AI Tools Used Across Included Studies and Their Performance

First Author (year)	ML Type /Algorithm	ML algorithm family	Biomarker(s)	Type of Biomarker	Potential Clinical Feasibility	Model Performance (Top performing model if many are presented)
Shephard (2024) [22]	Shallow Neural Network	Supervised Learning	Morphological and spatial nuclear features	Histopathological (image-derived)	High - Fully automated and explainable model; externally validated	**Internal Validation**: - AUROC = 0.77 (SD 0.08) - Recall (Sensitivity) = 0.84 (SD 0.07) **External Validation**: **-** AUROC = 0.75 (SD 0.01) - Recall (Sensitivity) = 0.92 (SD = 0.04)
Mahmood (2023) [21]	Multivariable logistic regression	Supervised Learning	Nuclear circularity, cytoplasmic eosin, epithelial perimeter (morphological)	Histopathological (image-derived)	Moderate - Requires digital pathology setup	Model 8 (7digital histological features + Binary Grade): **Training** : AUROC: 0.86 ; 95% CI: 0.77–0.96; *p* < 0.0001) **External Validation** : AUROC: 0.79; 95% CI: 0.70–0.87; *p* < 0.0001)
Liu (2017) [37]	Random Forest (Peaks-Random Forest)	Supervised Learning	DNA index values from exfoliative cells	Genetic	High – non-invasive test with fully explainable model	High-risk group (OCRI2 ≥ 0.5): 36.4% (4/11) developed OSCC Low-risk group (OCRI2 < 0.5): 5.3% (3/57) developed OSCC
Shams (2017) [33]	Deep Learning (Fisher Discriminant Analysis)	Supervised Learning	29 gene expression profiling (from Probeset dataset)	Genetic	Low - Requires gene expression profiling and ML expertise	-Using leave-one-out cross-validation, 96.5% average accuracy, -Sensitivity: 98.1% (for MT) - Specificity: 94.2% (for non-MT)
Jing (2022) [32]	12 types of supervised ML algorithms including XGBoost, KNN, and Neural Nets, among others	Supervised Learning	7-gene panel (DHX9, BCL2L12, RAD51, MELK, CDC6, ANLN, KIF4A)	Genetic	Low - Requires gene expression profiling and ML expertise	SVM algorithm: Test set AUROC = 0.75
Ellis (2022) [36]	PCA-LDA	Unsupervised (PCA) and Supervised (LDA)	Infrared absorbance spectra obtained via Fourier-transform infrared micro-spectroscopy	Biochemical Modality	Low – expensive and high-end research instruments and technical expertise required	-Sensitivity: 79 ± 4.9% in predicting MT -Specificity: 76 ± 5.1% in predicting MT
Hwang (2017) [30]	Straticyte proprietary algorithm (Cox regression model and cutoff selection steps)	Supervised Learning	S100A7 protein	Protein (IHC-based)	High - IHC based, quantitative image analysis	- Sensitivity (low-risk vs. intermediate- and high-risk Straticyte groups): 95% - NPV (low-risk vs. intermediate- and high-risk Straticyte groups): 78% - AUROC: 0.68 (compared to 0.67 for dysplasia grading) - C-index: 0.70 (compared to 0.67 for dysplasia grading)
Darling (2023) [34]	Straticyte proprietary algorithm (Cox regression model and cutoff selection steps)	Supervised Learning	S100A7 protein	Protein (IHC-based)	High - IHC accessible, high interobserver agreement	- Inter-observer agreement K = 0.89 Significant prediction of transformation (Assessor 1 - *p* = 0.047 and Assessor 2 - *p* = 0.030)
Bashir (2023) [35]	Deep Learning (Weakly supervised WSI model)	Weakly Supervised Learning	Peri-epithelial lymphocytes, epithelial/basal layer nuclei counts	Histopathological (image-derived)	Moderate - Requires deep learning setup, digital slides	Model: IDaRS (weakly supervised deep-learning pipeline) Performance: AUROC 0.78 ± 0.07, F1 (A measure used for uneven datasets to determine accuracy) 0.69 ± 0.0
Ali (2021) [31]	Custom algorithm, semi-automated image analysis	Unsupervised Learning	Invadopodia markers (Cortactin, Tks5, MMP14)	Protein (IHC-based)	Moderate - Requires FIHC and image analysis software	Model / Score: Invadopodia score (marker colocalization) performance (all samples): - Sensitivity = 75% (95% CI: 0.62–0.85) - Specificity = 76% (95% CI: 0.62–0.86) - AUROC = 0.83

*ML* Machine Learning, *AUROC* Area Under the Receiver Operating Characteristic curve, *KNN* k-Nearest Neighbors, *SVM* Support Vector Machine, *IHC* Immunohistochemistry, *NPV* Negative Predictive Value, *C-index* Concordance Index, *WSI* Whole Slide Image, *IDaRS* Iterative Draw-and-Rank Sampling, *FIHC* Fluorescent Immunohistochemistry, *PCA* Principal Component Analysis, *LDA* Linear Discriminant Analysis

### Technical specifications and digital pathology workflows

The technical workflows for the digital pathology and genomic studies demonstrated significant heterogeneity in image acquisition and data processing (Table 3). Scanner hardware varied across studies, with some utilizing multiple platforms to facilitate cross-site validation, while others relied on a single scanner type. Magnification levels for analysis ranged from x10 to x40 objectives. Spectroscopy-based studies, however, employ FTIR microscopy platforms acquiring hyperspectral data at 4 cm⁻¹ resolution with ~ 5–6 μm spatial sampling, requiring specialized detectors and atmospheric control systems. Regarding image preprocessing, only two studies explicitly reported the use of stain normalization or augmentation techniques to mitigate domain shifts [22, 35]. In contrast, FTIR workflows applied signal-processing pipelines including spectral smoothing, differentiation, paraffin-region exclusion, normalization, and dimensionality reduction prior to classification. Methodological approaches for image input also differed: four studies utilized manual region of interest selection or Tissue Microarrays [21, 30, 31, 34], whereas two employed Whole Slide Imaging workflows using Iterative Draw-and-Rank Sampling for automated tile selection [22, 35]. Annotation requirements varied from fully manual delineation of the epithelium to semi-automated segmentation using pre-trained networks like HoVer-Net. Artifact handling was generally addressed through the exclusion of tissue with folds, blurring, or tangential cuts, although specific criteria for these exclusions were inconsistently reported across the included studies.

**Table 3:** Pre-analytic and Technical Details Overview

First Author (Year)	Scanner & Magnification	Stain Normalization	Input Type & Tile Strategy	Annotation Requirements	Artifact Handling
Shephard (2024) [22]	**Scanner**: NanoZoomer 360, Aperio CS2, Pannoramic 250, Aperio AT2 **Mag**: Scanned ×40; Tiles ×20 (0.50*mpp*)	**Augmentation**: Used stain augmentation (Macenko method) to counter domain shifts	**WSIs: IDaRS** (*k* = 5 top predictive, *r* = 45 random patches). Tessellated with 50% overlap.	**Semi-Automated**: HoVer-Net+ trained on manually delineated layers and point-annotated nuclei	**Excluded**: Slides not meeting study criteria (e.g., tangential cuts implied)
Mahmood (2023) [21]	**Scanner**: Aperio CS2, NanoZoomer 360, Pannoramic 250, Aperio AT2 **Mag**: Cell detection at ×10	-	**ROIs**: Manual selection of 3–4 ROIs per WSI (300,000±400* μm*).	**Manual**: Full thickness of epithelium demarcated within ROIs.	**Excluded**: Slides with distortion, blurring, tissue artifacts, or folds.
Liu (2017) [37]	NA (Genetic Data)	NA	Engineered, 1-dimensional integer vector of 10 units long, representing tallied “peaks” across 10 specific intervals based on cell ploidy	NA	NA
Shams (2017) [33]	NA (Genomic Data)	NA (Batch effect removal performed)	**Genomic Data: g**ene expression profiles	NA	NA
Jing (2022) [32]	NA (Genomic Data)	NA (Batch effect removal performed)	**Genomic Data**: mRNA expression profiles from GEO/TCGA databases.	NA	NA
Ellis (2022) [36]	**Scanner**: Varian 620 microscope coupled to an Agilent Cary 670 **Mag**: -	**None.** FTIR data was collected from routine, unstained histopathological sections	Highly dimensional hyperspectral **IR images**, with **built-in** mosaic functions	**Manual: S**pecialist head and neck histopathologist manually marked the most extreme area of dysplasia	**Excluded**: PCA and Hotelling’s T² summary statistic to identify and discard any anomalous spectra falling outside the 95% confidence interval
Hwang (2017) [30]	**Scanner**: Hamamatsu Nanozoomer-XR **Mag**: ×20	**-**	**ROIs & Tissue Microarrays**: Manual selection of three 300-*µm* diameter ROIs.	**Manual**: ROIs selected by 2 independent observers.	**Excluded**: Cells from folded, out-of-focus tissues.
Bashir (2023) [35]	**Scanner**: Aperio CS2 (×20) & Hamamatsu Nanozoomer (×40) **Mag**: Patches extracted at 0.50* μm*/*px* (≈×20),	**Yes.** Input patches stain-normalized using a TCGA sample.	**WSIs: IDaRS** (Iterative Draw-and-Rank Sampling). Selects random (*r* = 30) and top-ranked (*k* = 5) patches.	**Semi-Automated**: Epithelium masks via HoVer-Net+ refined manually. Slide-level labels used.	**Excluded**: Tangentially cut sections and tissue with artefacts.
Darling (2023) [34]	**Scanner**: Hamamatsu Nanozoomer-XR **Mag**: ×20	**-** (IHC for S100A7)	**WSIs**: Images imported into Visiopharm VIS; likely ROI-based following Hwang et al. methodology	**Manual**: Assessment by two independent assessors blinded to clinical info.	-
Ali (2021) [31]	**Scanner**: Quorum Spinning Disk Confocal microscope **Mag**: ×10 objective, ×1.6 lens	**None.** (Correction factor applied for cell density/size)	**ROIs**: 10 static image fields randomly selected per slide using X, Y coordinates to prevent overlap.	**Manual**: Operator manually selected the epithelium in images.	**Excluded**: Cases removed due to poor quality sections; requires “undisturbed epithelium”

*FTIR* Fourier-transform infrared, *IDaRS* Iterative Draw-and-Rank Sampling, *IHC* Immunohistochemistry, *Mag* Magnification, *NA* Not applicable, - not reported, *PCA* Principal Component Analysis, *ROI* Region of Interest, *WSI* Whole Slide Image

### Machine learning approaches

Although studies employed varying biomarkers, their AI/ML approaches represented an even greater heterogeneity. Within the realm of ML algorithms, three main approaches exist, namely supervised, weakly supervised, and self-supervised learning. Each approach carries intrinsic advantages and disadvantages and choosing between approaches hinges on the amount of available data, its quality, the required level of model interpretability, and the trade-off between data annotation efforts and predictive performance of models.

Supervised learning was the most frequently used strategy, employed in eight of the ten included studies, including those by Shephard et al., Mahmood et al., Jing et al., Darling et al., Hwang et al., Shams et al., Liu et al., and Ellis et al. [21, 22, 30, 32–34, 36, 37]. These models relied on labeled datasets linking biomarker features to clinical outcomes. While supervised learning demonstrated strong predictive performance across modalities, its implementation required extensive labeled data and introduced potential risks of overfitting when validation strategies were insufficient. Weakly supervised learning was used exclusively by Bashir et al., who implemented an Iterative Draw-and-Rank Sampling pipeline to predict progression-free survival from histologic images [35]. Weakly supervised models learn from partial, imperfect, or noisy data to predict the correct output [39]. This model’s performance demonstrated the feasibility of training models using partially labeled data. Self-supervised or unsupervised learning approaches were limited among included studies. Ali et al. employed a semi-automated rule-based algorithm generating an Invadopodia score from fluorescence immunohistochemistry data [31]. This model’s performance also demonstrated strong predictive capability despite reduced reliance on labeled training data. Overall, supervised learning represented the dominant strategy, while weakly supervised and self-supervised methods were less frequently used but demonstrated promising performance in settings with limited labeled data.

### Machine learning model performance

To perform a structured comparison of heterogeneous methodologies, we reported the models’ performance stratified by biomarker categories mentioned previously: morphological, protein-based, genetic, and biochemical.

Morphological biomarker models demonstrated consistently strong predictive performance across multiple studies. These models relied on quantitative histologic image features extracted from hematoxylin and eosin (H&E) stained slides. Shephard et al. implemented a fully automated two-stage AI pipeline using a HoVer-Net+ deep-learning model to segment nuclei and epithelial layers. This was followed by extraction of interpretable morphological and spatial features that were processed through a shallow neural network to generate an Oral Malignant Transformation Score [22]. On external validation, the model achieved an AUROC of 0.75 with a recall of 0.92, outperforming binary grading (AUROC = 0.72; recall = 0.85). Internal validation yielded an AUROC of 0.77, exceeding both WHO (0.68) and binary grading (0.71), and demonstrated strong prognostic performance with a C-index of 0.74 (*p* < 0.001). Mahmood et al. developed multivariable logistic regression models using quantitative histologic features including epithelial cellularity, nuclear circularity, nuclear eccentricity, optical density metrics, and nuclear-to-cytoplasmic ratios [21]. Their primary model achieved an AUROC of 0.77 (95% CI 0.65–0.90), outperforming WHO grading (0.68). Augmenting the model with WHO or binary grades increased performance to AUROC 0.85 and 0.86, respectively. External validation demonstrated robust performance (AUROC 0.76; 95% CI 0.68–0.85). Bashir et al., who trained their models based on lymphocytes and nuclei counts, achieved an AUROC of 0.78 (± 0.07 SD) [35]. Overall, morphological models demonstrated AUROC values ranging from approximately 0.75 to 0.86, representing the highest and most consistently externally validated performance among included modalities.

Protein-based models utilized immunohistochemical markers to quantify biologically relevant protein expression patterns associated with MT. Hwang et al. and Darling et al. implemented the Straticyte algorithm, which integrates cytomorphometric features with quantitative S100A7 staining intensity to generate individualized risk scores using multivariate Cox regression [30, 34]. The method demonstrated high reproducibility and strong predictive performance, with 65% of elevated-risk lesions progressing compared to 0% of low-risk lesions, outperforming conventional dysplasia grading systems. Ali et al. developed an Invadopodia score based on fluorescence immunohistochemistry detection of Cortactin, Tks5, and MMP14 colocalization [31]. The model achieved sensitivity of 75%, specificity of 76%, and an AUROC of 0.82, demonstrating reliable prediction of progressing lesions. Across studies, protein-based models demonstrated AUROC values ranging from approximately 0.76 to 0.82, with strong biological interpretability but variable validation strategies.

Genetic models used gene-expression profiling combined with machine learning classifiers to predict MT risk. Jing et al. evaluated 12 supervised machine-learning algorithms to construct multi-gene risk models [32]. All models achieved validation AUROC values greater than 0.65, with the Support Vector Machine model achieving the highest AUROC of 0.749. The naïve Bayes model demonstrated stable performance between training and validation sets (AUROC 0.728 vs. 0.711), while random forest models exhibited signs of overfitting (training AUROC = 0.999; validation AUROC = 0.725). Shams et al. utilized Fisher Discriminant Analysis for gene selection followed by deep-learning classification [33]. Their model demonstrated high predictive performance, achieving 96.5% accuracy and 98.1% sensitivity using leave-one-out cross-validation. Liu et al. developed the OCRI2 index using Peaks-Random Forest modeling of DNA-index measurements from exfoliative cytology [37]. Their model identified a high-risk cohort in which 36.4% of patients developed oral cancer, significantly outperforming conventional cytologic methods (*p* = 0.01). Overall, genetic models demonstrated AUROC values typically ranging from approximately 0.71 to 0.75. Performance variability was observed, however, due to methodological differences and risks of feature-selection bias.

Biochemical approaches relied on spectral or chemical profiling methods to identify transformation-associated molecular signatures. Ellis et al. implemented a Principal Component Analysis followed by Linear Discriminant Analysis pipeline to analyze FTIR spectra obtained from epithelial tissues [36]. This biochemical model predicted malignant transformation with sensitivity of 79 ± 4.9% and specificity of 76 ± 5.1%, outperforming standard histopathological grading within their pilot cohort. Biochemical approach demonstrated moderate predictive performance, with sensitivity and specificity values generally ranging between 76% and 79%. However, this was a pilot study with a small cohort.

### Algorithmic rigor and validation strategies

The studies exhibited significant variation in their handling of data leakage and feature selection bias (Table 4). Shephard et al. and Mahmood et al. demonstrated robust methodology by validating their models on fully independent external cohorts, while Hwang et al. rigorously prevented leakage during internal validation by recompiling the algorithm and cut-offs within each training fold [21, 22, 30]. Conversely, Jing et al. introduced a high risk of leakage by performing batch effect removal and gene selection on the entire pooled dataset prior to cross-validation. The other genomic studies (Shams et al. and Liu et al.) are also susceptible to a high risk of leakage as gene selection was done prior to cross-validation. Ali et al. may have inflated diagnostic accuracy by optimizing threshold cut-offs (Youden index) on the same full dataset used for testing [31]. Bashir et al. mitigated some risks through external stain normalization and stratified cross-validation but lacked independent external validation [35]. The study by Ellis et al., while lacking an independent external cohort, employed a leave-one-pair-out cross-validation which ensures the preprocessing optimization and model training were confined strictly to each training fold. Equal spectral sampling per lesion was additionally implemented to minimize sample-related bias [37]. Finally, Darling et al. served as a validation study of an existing assay, rendering training-specific biases irrelevant [34].

**Table 4:** Methodological Rigor of Machine Learning Workflows: Feature Selection and Validation Strategies

First Author (Year)	Segmentation & Normalization	Hyperparameter Tuning Strategy	External Validation Independence
Shephard (2024) [22]	Automated (HoVer-Net+); used stain augmentation	Thresholds set via internal cross-validation before external testing	High: Independent sites (Belfast/Birmingham), different scanners & staining
Mahmood (2023) [21]	Quantitative Histology on manual ROIs	Tested 8 feature combinations on the development cohort	High: Multi-center (UK/Brazil) with 4 different scanner types
Liu (2017) [37]	N/A: Raw data consisted of DNA index values obtained from exfoliative cells	Modeling performed using the Caret package in R (default parameters) combined with a 10-fold cross-validation	None: Single patient population, no independent cohort
Shams (2017) [33]	N/A (Genomic); Raw microarray data used; Fisher Discriminant Analysis for feature selection	DNN: 10-fold cross-validation to adjust the number of nodes in each layer Multi-layer perceptron: 10-fold cross-validation used to set the optimal values for the number of nodes and the number of iterations Regularized Least Squares: The optimal value for the regularized parameter determined by leaving one sample out of cross-validation for the training set	None: Single dataset of 86 patients accessed via NCBI GEO
Jing (2022) [32]	Batch effects removed from pooled datasets (potential leakage)	10-fold cross-validation for 12 algorithms; nested tuning loops not specified	None: Pooled public datasets; no independent clinical OPMD cohort.
Ellis (2022) [36]	Manual ROI selection	Optimization framework to determine the best pre-processing steps and model hyperparameters	None: Model evaluated internally using a leave-one-pair-out cross-validation strategy
Hwang (2017) [30]	Manual ROI selection	100x split sample (110 train/40 test); cut-offs recompiled per iteration	None: Single retrospective study; calls for independent cohorts
Bashir (2023) [35]	Automated with manual refinement; stain-normalization.	IDaRS hyperparameters set for experiments; separate tuning set not specified.	None: Unicentric cohort (Sheffield); requires multi-center data.
Darling (2023) [34]	Applied pre-existing Straticyte algorithm	NA (Validated pre-defined risk classes)	Partial: Validation of a previous method on an independent UK biobank
Ali (2021) [31]	Semi-automated; manual epithelium selection	Invadopodia score cut-offs calculated on the entire dataset (Youden index)	None: Retrospective case-control from a single center (Toronto)

*DNN* Deep neural network, *IDaRS* Iterative Draw-and-Rank Sampling, *NA* Not applicable, *ROI* Region of interest

## Discussion

In this study, we synthesized available evidence on prognostic models integrating AI/ML and biomarkers to provide insight into the risk of progression from OPMD to OSCC. Although this approach shows promise for improving risk stratification, our QUADAS-2 assessment identified a critical “validation gap” that may limit clinical translation. A high risk of bias in the Patient Selection and Flow and Timing domains suggests that reported performance metrics may be inflated due to spectrum bias related to retrospective designs and inconsistent follow-up. Furthermore, the ‘Unclear’ risk ratings associated with proprietary tools such as Straticyte underscore the need for greater technical transparency. Notably, all included models were developed and evaluated using retrospective datasets, leaving their performance and clinical utility in real-world settings uncertain.

### Heterogeneity in biospecimens and analytical platforms: implications for clinical deployability

The main contributor to the methodological heterogeneity is differences in required inputs and infrastructure (Table 5). Histomorphological features offer lower pre-analytic costs, rapid turnaround, and rely mainly on standard H&E slides and digital scanners already available in many pathology laboratories, making their clinical deployability more favorable.

**Table 5:** Comparison of Biomarker Categories and Their Clinical Deployability

Modality	Cost	Turnaround Time	Infrastructure Needs	Batch Effects / Variability	Clinical Readiness
H&E morphology	Low	Fast (hours to days)	Digital scanner and AI pipeline	Sensitive to staining, scanner differences	Widely available infrastructures in pathology labs
IHC protein biomarkers	Moderate	Moderate (days)	Antibodies, standardized staining lab	Antibody variability, scoring subjectivity	Infrastructures already in use for clinical purposes
Bulk gene expression panels	High	Slower (days to weeks)	Sequencing, molecular lab and bioinformatics	Strong batch effects, RNA quality dependence	Limited by cost and access
Biochemical modalities	Moderate	Fast (hours to days)	Specialized laboratory equipment, including a microscope coupled to a spectrometer.	Very sensitive to the amount of substance being probed. Does not require staining	Early stage with high potential as an alternative to Immunohistochemistry

*H&E* Hematoxylin and Eosin, *IHC* Immunohistochemistry, *AI* Artificial Intelligence

Protein-based biomarkers and gene expression panels are generally more costly and time-intensive. However, protein assays integrate relatively well into existing clinical workflows due to an established staining platform. Nonetheless, all modalities are vulnerable to batch effect. Gene expression panels are highly sensitive to RNA quality. On the other hand, protein assays may vary due to antibody performance and scoring subjectivity, while histomorphic approach can be influenced by differences in scanners and staining protocols if not standardized. Biochemical-based studies are still in their early stages; however, they show high potential, as the analysis can be performed on unstained histopathological sections, thus eliminating the complex staining protocol. Additionally, each small region contains many particles that can absorb infrared light, providing massive volumes of data for ML models to analyze.

### Clinical and epidemiologic sources of heterogeneity

Although the included studies pursued similar objectives, their direct comparison is not feasible due to substantial heterogeneity. Differences in demographic reporting limit cross-study comparisons and highlight the need for standardized demographic data collection in future prognostic OPMD research. Despite this, most studies reported a male predominance which is consistent with evidence that men are diagnosed with oral cancer, including OPMDs and OSCC, at higher rates than women [40–42]. Studies examining risk factors indicate that traditional exposures such as smoking, alcohol, and smokeless tobacco contribute to these differences [40]. Heterogeneity in grading systems and lesion distribution underscores the variability inherent in current OPMD prognostic research. The anatomical distribution of OPMDs in the included studies revealed a significant bias toward tongue lesions. This site-specific dominance is a critical factor for clinical applicability, as different oral mucosal regions exhibit distinct biological behaviors and baseline transformation rates, with research demonstrating that lesions on the tongue can carry a hazard ratio for transformation 2.41 times higher than that of buccal lesions [43]. Consequently, AI/ML models trained predominantly on tongue lesions may incorrectly estimate risk when applied to other anatomical sites, as etiological drivers and progression kinetics may differ. To ensure clinical reliability, future algorithm research should utilize site-balanced datasets or explicitly incorporate anatomical location as a weighted feature within the algorithm. Most included studies failed to account for or match participants for key clinical covariates, such as anatomical location, smoking, alcohol, HPV status, and surveillance intensity, which confound results. Only Ali et al. performed partial matching based on diagnosis and location, while Mahmood et al. excluded HPV-related lesions, but did not account for other variables [21, 31]. This lack of covariate control may artificially inflate model performance by capturing imbalanced clinical risk factors rather than true transformation-specific biological signals. Finally, follow-up and study periods were different between studies. This inconsistency in follow-up duration and reporting hindered meaningful direct comparisons across studies.

### Model performance and validation considerations

The included AI/ML models demonstrated variable predictive performance, and while some metrics appear encouraging, these must be interpreted with caution given the methodological concerns identified in this review. Most models lacked external validation, were developed on small retrospective cohorts, and several carry a high risk of data leakage (Table 4), all of which are known to produce optimistic AUROC estimates that may not generalize to clinical populations. AUROC values for predicting MT ranged from 0.74 to 0.78, with some models reporting high sensitivity or recall but lower specificity, or vice versa. This variation highlights differences in model architecture, biomarkers used, cohort characteristics, and outcome definitions. Most studies used AUROC for performance reporting which offers benefits compared to threshold-based metrics such as sensitivity and specificity but carries some drawbacks. AUROC reflects the model’s ability to discriminate between OPMDs that progress to OSCC and those that do not, across all possible classification thresholds. This feature makes AUROC useful for comparing prognostic models in early development. However, AUROC alone cannot guide clinical decision-making at a specific operating threshold. In contrast, the threshold-based metrics measure performance at a chosen cut-off thereby making these values more clinically interpretable. We believe that while AUROC and threshold-based metrics are complementary and should consistently be reported, the complex process of MT makes selecting a clear cut-off a difficult task.

A critical and underappreciated threat to the validity of the reported performance metrics across included studies is the risk of data leakage, which is the inadvertent incorporation of test-set information into model training, most commonly through feature selection, batch correction, or threshold optimization performed on the full dataset prior to cross-validation. When leakage occurs, reported AUROC and sensitivity values reflect the model’s ability to memorize patterns in a single dataset rather than its capacity to generalize to previously unseen cases, rendering such metrics less clinically informative. To address this within the present review, we categorized the included studies by their overall leakage risk. Five studies were judged to be at low risk, including those from Shephard et al., Mahmood et al., Hwang et al., Ellis et al., and Darling et al. Bashir et al.’s study was the only one with moderate risk. The remaining four studies were judged to be at high risk, as gene selection, batch correction, or threshold optimization were performed on the full dataset prior to any validation step. Taken together, nearly half of the included studies carry a high risk of leakage, meaning that their reported metrics should be treated as upper-bound estimates rather than reliable indicators of real-world predictive performance. Future OPMD prognostic studies must adhere to strict data partitioning protocols, including nested cross-validation for all preprocessing and feature selection steps, and report these details with sufficient transparency to allow independent appraisal of leakage risk.

Three models included external validation, a crucial step for assessing generalizability and building confidence in model performance. Of note, external validation, or lack thereof, is argued to be neither sufficient nor required as an essential step before implementation. According to a recent paper by la Roi-Teeuw et al., external validation is not necessarily a stronger indicator of model performance, nor does it prove the model’s generalizability or represent the final step before clinical implementation [44]. Therefore, future models should be accompanied by clear documentation of their intended use, as well as ongoing processes for model updating and validation [44]. However, we believe that prospective validation will allow clinicians and patients to evaluate models in real-world conditions rather than just on historical datasets, which could greatly aid their approval and adoption. A significant finding of this review is the lack of standardized reporting across the included studies. None of the identified papers explicitly utilized established reporting frameworks such as Transparent Reporting of a multivariable prediction model for Individual Prognosis Or Diagnosis (TRIPOD-AI) or Checklist for Artificial Intelligence in Medical Imaging (CLAIM) [45, 46]. This lack of transparency makes it difficult to fully validate reported performance metrics, assess risk of bias, or ensure reproducibility. Future OPMD research must strictly adhere to these reporting guidelines to ensure methodological rigor, facilitating an efficient transition from exploratory research to actionable clinical applications and aiding in the head-to-head model comparisons.

### Technical landscape: type of AI/ML and biomarker inputs

From a technical perspective, Jing et al. and Mahmood et al. used more traditional algorithms such as multivariable logistic regression and ensemble methods, whereas Shephard et al. and Ali et al. used deep learning approaches, including shallow neural network and weakly supervised convolutional neural network [22, 31]. While supervised and weakly-supervised models were present in all but one study, unsupervised learning, utilized by Ali et al. through a rule-based approach, represented a clinically valuable methodology. The model achieved an AUROC of 0.83 without the need for the large, labeled training datasets required by the other included studies. This approach avoided the data labeling bottleneck often tied to the building of supervised and weakly-supervised prognostic models, while providing a superior biological interpretability compared to traditional “black-box” supervised models. These attributes suggest its application in different clinical settings or emerging research niches where high-quality, expert-labeled training data are scarce.

Biomarker selection played a critical role in the performance of AI/ML models for assessing MT risk in OPMDs. One of the promising candidates, S100A7, has been consistently associated with early-stage and well-differentiated OSCC, but not with late-stage or poorly differentiated tumors [47–49]. Mechanistic studies have shown that S100A7 promotes tumor progression through activation of the p38/MAPK pathway. This pathway upregulates invasive markers like RAB2A, and modulates apoptosis and adhesion proteins, thereby enhancing cancer cell survival, migration, and invasion [16, 48, 50]. Similarly, Invadopodia, which are actin-rich protrusions implicated in extracellular matrix degradation, have also emerged as biomarkers that could contribute to cancer invasion [48]. Their formation is driven by proteins such as Tks5, cortactin, and MMP-14. These proteins are regulated by EGFR/Src signaling and the tumor microenvironment, including factors secreted by cancer-associated fibroblasts and cytokines like TNF-α and IL-8 [51, 52].

In addition to protein-based markers, gene expression profiling offers valuable insights into MT. The Cancer Genome Atlas identified recurrent mutations in oncogenes and tumor suppressors such as TP53, PIK3CA, and CDKN2A in OSCC [53–55]. Recent studies advocate for the use of multimarker gene panels over individual biomarkers due to their superior predictive accuracy as they capture the molecular heterogeneity of OSCC. Importantly, non-invasive sampling methods such as saliva collection make genomic risk assessment more clinically feasible for early detection and longitudinal monitoring [32, 53, 54].

A promising biochemical biomarker candidate for OSCC MT prediction is FTIR spectroscopy. FTIR provides an objective chemical “fingerprint” of tissues that correlates with established biological indicators of MT, such as DNA aneuploidy, glycogen depletion, and protein structural alteration [56]. This biomarker relies on hyperspectral maps that do not require staining. Therefore, FTIR could offer a cost-effective and less subjective alternative to conventional histopathological grading. FTIR is in its early stages of research in OPMD risk stratification and future prospective studies are required to further validate and test this emerging modality.

Another notable finding in this review is the superior predictive performance of image-derived morphological features compared to individual molecular biomarkers. We hypothesize that morphological features act as a phenotypic proxy, aggregating the downstream, cumulative effects of multiple genetic and epigenetic alterations into stable, quantifiable visual signals. In this way, morphological and spatial features may predict progression more reliably than conventional grading [21, 22]. While single modality molecular biomarkers capture isolated biological events, morphological changes represent the final product of diverse oncogenic drivers. Furthermore, digital pathology models can integrate the spatial relationship between the epithelium and the surrounding stroma, capturing critical microenvironmental factors such as peri-epithelial lymphocyte activity that purely molecular assays may overlook [35]. Therefore, the phenotypic summation of many pathological processes, which can be observed through morphological features, likely provides a more robust and generalizable signal for machine learning algorithms.

A critical aspect of AI/ML training in this field is the reliance on histopathological assessment as the primary “ground truth,” or gold standard. This introduces two key limitations. First, OPMD grading is inherently subject to significant intra- and inter-observer variability and has limited reproducibility even among expert oral pathologists [10, 11]. When models are trained on these annotations, the supervised learning objective is not to predict biological transformation but rather to predict a human rater’s subjective grading decision. This creates a form of circularity: a model that achieves high performance may simply be learning to replicate inter-pathologist inconsistencies rather than capturing the true underlying biological drivers of MT. Second, histopathological grade is a noisy label for MT risk. As discussed in this review, dysplasia grade alone does not reliably predict transformation, mild dysplasia can progress while severe dysplasia may regress. Models are therefore trained on a label that is not itself a reliable predictor of the outcome it is intended to predict. To overcome these limitations, future studies should prioritize the use of hard clinical endpoints such as time-to-transformation or incorporate molecular transformation markers as more robust ground-truth labels. This shift would allow AI/ML models to move past these limitations and provide more accurate and clinically actionable risk predictions.

### Clinical relevance and future directions

One of the most compelling clinical opportunities offered by AI/ML-driven risk prediction tools is the potential to inform and personalize decision-making. OPMD management currently spans a spectrum from active surveillance to surgical intervention, with decisions often based on clinician judgment, conventional grading, and subjective assessment of progression risk.

Previous research has shown that OPMD excision negatively impacts patients’ quality of life [57]. Clinicians should prioritize safely ruling out MT while avoiding overtreatment, leveraging emerging technologies to aid in the triage of OPMD lesions and inform follow-up frequency for low- versus high-risk patients. Therefore, maximizing NPV should be a priority in future models, focusing on carrying out clustered randomized clinical trials to assess whether these tools improve decision-making, patient outcomes, and healthcare resource utilization.

To translate AI/ML-based prognostic tools from research to clinical settings, a structured implementation framework is needed. From an infrastructure standpoint, digital pathology-based models require whole-slide imaging scanners, secure data storage, and AI inference pipelines integrated with laboratory information systems, which are resources that remain unavailable in many community and resource-limited settings. Genomic and gene expression-based approaches additionally require access to sequencing platforms, RNA-quality-controlled biobanks, and bioinformatic expertise, substantially limiting their near-term deployment outside academic centers. IHC-based approaches are more immediately deployable as they leverage existing staining infrastructure but require standardization of antibody platforms and scoring protocols across sites. From a clinical workflow perspective, AI/ML tools would ideally be integrated as decision-support adjuncts at the time of biopsy reporting, providing a risk stratification output alongside the pathologist’s grading to guide surveillance frequency and intervention decisions. This requires bidirectional integration with electronic health records and pathology reporting systems. From a regulatory standpoint, AI/ML medical devices in Canada fall under Health Canada’s Software as a Medical Device (SaMD) framework, while in the United States the FDA’s Digital Health Center of Excellence oversees AI/ML-based SaMD pathways [58, 59]. Developers of tools like Straticyte must provide prospective clinical evidence of patient benefit, not merely technical performance, to meet the evidentiary standards required for approval and reimbursement. Future research should explicitly address these deployment requirements as part of the study design.

To advance the transitional potential of biomarker and AI/ML-driven risk stratification in OPMD, several novel research avenues warrant exploration. First, the majority of current studies develop AI/ML models that rely on a single modality input. Integration of a panel of biomarkers within a single predictive model has the potential to reveal a more nuanced and accurate pattern of progression and recurrence. Second, none of the studies assessed the utility of AI/ML or evaluated its performance in a prospective manner within a clinical setting. While retrospective studies allow for validation and development of these models, prospective studies provide a perspective concerning implementation of AI/ML tools in clinical workflow, its impact on shared decision-making, and its cost-effectiveness. Third, AI/ML-based studies under-investigate the role of salivary biomarkers in prognostication of OPMDs, despite several studies establishing a correlation between various salivary biomarkers and malignant progression in OSCC [60, 61]. Given that OSCC develops through a multistep accumulation of genetic alterations, salivary time-series monitoring could effectively track OPMD progression toward MT. Salivary biomarkers have an advantage over others due to their convenience, non-invasiveness, and low cost, facilitating longitudinal monitoring of OPMDs, which could prove useful in remote, underserved communities. Additionally, researchers should leverage time-series models to capture OPMD evolution and identify the optimal window for surgical intervention. Moving beyond static, single-point data, we propose incorporating serial biopsies or salivary markers into algorithm training to better track disease progression toward MT. Lastly, different clinical subtypes of OPMDs could behave differently in the path towards MT. Therefore, future models should make a distinction between clinical OPMD subtypes.

### Implications for global oral health

The clinical utility of AI/ML-driven prognostic tools is ultimately dependent on their generalizability across diverse demographic and etiological landscapes. The included studies fall substantially short of this standard. A defining finding of this review is that none of the included studies incorporated patient cohorts from high-incidence regions where betel quid and areca nut chewing are prevalent, particularly South Asia and Southeast Asia. These habits are estimated to contribute to nearly 50% of the region’s oral cancer cases [3]. This represents an important etiological gap as betel quid-associated OPMDs exhibit distinct molecular phenotypes, carcinogenesis pathways, and progression kinetics compared to tobacco- and alcohol-associated lesions predominant in Western cohorts. Therefore, AI/ML models trained exclusively on Western cohorts may not be able to generalize to these populations, introducing an important algorithmic bias. Western-derived models may systematically underestimate or overestimate transformation risk, with direct consequences for patient triage and overtreatment.

Furthermore, the datasets used in the included studies reflect structural inequities in research funding and infrastructure. No cohort was assembled by engaging with community health systems or population-level registries in high-burden, low-resource settings. This creates a feedback loop in which populations heavily impacted by oral cancer are least represented in the tools being developed to manage it, exacerbating global health inequities. To address this, future studies should deliberately recruit from betel quid/areca nut-exposed cohorts, report ethnicity and cultural risk factor data as primary demographic variables and perform domain adaptation analyses to assess the generalizability and domain shift of trained models when applied to external population strata, moving beyond western-centric datasets. Ultimately, the promise of AI/ML-driven risk stratification can only be realized if the data underlying these models reflect the full global burden of OPMD. Failing to do so risks further entrenching existing health inequities into the clinical tools designed to address them.

## Conclusion

In conclusion, the available evidence suggests that integrating AI/ML with validated biomarkers has the potential to provide prognostic insights into the risk of MT in OPMDs. However, these tools require further validation and improvement before routine clinical use. The main limitations across all studies include the lack of prospective validation, inconsistent clinical and demographic reporting, and lesion site diversity, all of which negatively affect the generalizability and clinical applicability of the existing tools. Moving forward, future research should prioritize prospective clinical evaluation, multimodal biomarker integration, and inclusive cohort representation to ensure that future models can accurately stratify risk across diverse patient populations. Although prospective clinical trials are an essential next step, several feasibility barriers must be addressed first. Implementing image-based models requires major investment in digital pathology infrastructure. In many clinical settings the costs and technical expertise needed for digital H&E processing or genomic sequencing remain significant obstacles. Future research must assess the cost-effectiveness of these AI tools relative to standard histopathology to support their integration into routine practice. Addressing these concerns could refine surveillance strategies, optimize intervention timing, and ultimately reduce the burden of OSCC.

## Supplementary Information

Supplementary Material 1.

## Data Availability

All data underlying the results of this scoping review are derived from publicly available literature cited in the reference list. Supplementary materials including the extraction table can be provided, upon request, by the corresponding author.

## Abbreviations

AI: Artificial intelligence
AUROC: Area Under the Receiver Operating Characteristic curve
FTIR: Fourier–transform infrared
H&E: Hematoxylin and Eosin
IHC: Immunohistochemistry
IQR: Interquartile range
JBI: Joanna Briggs Institute
OSCC: Oral squamous cell carcinoma
OPMD: Oral potentially malignant disorder
ML: Machine learning
MT: Malignant transformation
NPV: Negative predictive value
PPV: Positive predictive value
QUADAS-2: Quality Assessment of Diagnostic Accuracy Studies
WHO: World Health Organization

## References

[1] Cancer CCS. June / S canadienne du. Survival statistics for oropharyngeal cancer. Canadian Cancer Society. https://cancer.ca/en/cancer-information/cancer-types/oropharyngeal/prognosis-and-survival/survival-statistics. Accessed 1 2025.

[2] Wang B, Zhang S, Yue K, Wang X-D. The recurrence and survival of oral squamous cell carcinoma: a report of 275 cases. Chin J Cancer. 2013;32:614–8. 10.5732/cjc.012.10219. 23601241 10.5732/cjc.012.10219 PMC3845544

[3] Warnakulasuriya S. Global epidemiology of oral and oropharyngeal cancer. Oral Oncol. 2009;45:309–16. 10.1016/j.oraloncology.2008.06.002. 18804401 10.1016/j.oraloncology.2008.06.002

[4] Hoene G, Gruber RM, Leonhard JJ, Wiechens B, Schminke B, Kauffmann P, et al. Combined quality of life and posttraumatic growth evaluation during follow-up care of patients suffering from oral squamous cell carcinoma. Mol Clin Oncol. 2021;15:189. 10.3892/mco.2021.2351. 34349989 10.3892/mco.2021.2351 PMC8327079

[5] Liao C-T, Lee L-Y, Huang S-F, Chen I-H, Kang C-J, Lin C-Y, et al. Outcome analysis of patients with oral cavity cancer and extracapsular spread in neck lymph nodes. Int J Radiat Oncol Biol Phys. 2011;81:930–7. 10.1016/j.ijrobp.2010.07.1988. 20934267 10.1016/j.ijrobp.2010.07.1988

[6] Organisation mondiale de la santé. Centre international de recherche sur le cancer. editors. WHO Classification of Head and Neck Tumours. 4th ed. Lyon: International agency for research on cancer; 2017.

[7] McCord C, Achita P, Kiss A, Magalhaes MA, Darling M, Bradley G. Progression to malignancy in oral potentially malignant disorders: a retrospective study of 5,036 patients in Ontario, Canada. Oral Surg Oral Med Oral Pathol Oral Radiol. 2023;136:466–77. 10.1016/j.oooo.2023.06.006. 37563059 10.1016/j.oooo.2023.06.006

[8] Speight PM, Khurram SA, Kujan O. Oral potentially malignant disorders: risk of progression to malignancy. Oral Surg Oral Med Oral Pathol Oral Radiol. 2018;125:612–27. 10.1016/j.oooo.2017.12.011. 29396319 10.1016/j.oooo.2017.12.011

[9] Dey KK, Bharti R, Dey G, Pal I, Rajesh Y, Chavan S, et al. S100A7 has an oncogenic role in oral squamous cell carcinoma by activating p38/MAPK and RAB2A signaling pathway. Cancer Gene Ther. 2016;23:382–91. 10.1038/cgt.2016.43. 27767088 10.1038/cgt.2016.43

[10] Reibel J. Prognosis of oral pre-malignant lesions: significance of clinical, histopathological, and molecular biological characteristics. Crit Rev Oral Biol Med Off Publ Am Assoc Oral Biol. 2003;14:47–62. 10.1177/154411130301400105. 10.1177/154411130301400105 12764019

[11] Yan F, Reddy PD, Nguyen SA, Chi AC, Neville BW, Day TA. Grading systems of oral cavity pre-malignancy: a systematic review and meta-analysis. Eur Arch Oto-Rhino-Laryngol Off J Eur Fed Oto-Rhino-Laryngol Soc EUFOS Affil Ger Soc Oto-Rhino-Laryngol-Head Neck Surg. 2020;277:2967–76. 10.1007/s00405-020-06036-1 10.1007/s00405-020-06036-1 32447493

[12] Prasad G, Seers C, Reynolds E, McCullough MJ. A panel of microRNAs can be used to determine oral squamous cell carcinoma. J Oral Pathol Med Off Publ Int Assoc Oral Pathol Am Acad Oral Pathol. 2017;46:940–8. 10.1111/jop.12592. 10.1111/jop.12592 28512746

[13] de Jong EP, Xie H, Onsongo G, Stone MD, Chen X-B, Kooren JA, et al. Quantitative proteomics reveals myosin and actin as promising saliva biomarkers for distinguishing pre-malignant and malignant oral lesions. PLoS ONE. 2010;5:e11148. 10.1371/journal.pone.0011148. 20567502 10.1371/journal.pone.0011148 PMC2887353

[14] Fillies T, Woltering M, Brandt B, Van Diest J-P, Werkmeister R, Joos U, et al. Cell cycle regulating proteins p21 and p27 in prognosis of oral squamous cell carcinomas. Oncol Rep. 2007;17:355–9. 17203174

[15] de Vicente J-C, Lequerica-Fernández P, Santamaría J, Fresno M-F. Expression of MMP-7 and MT1-MMP in oral squamous cell carcinoma as predictive indicator for tumor invasion and prognosis. J Oral Pathol Med Off Publ Int Assoc Oral Pathol Am Acad Oral Pathol. 2007;36:415–24. 10.1111/j.1600-0714.2007.00546.x. 10.1111/j.1600-0714.2007.00546.x 17617835

[16] Hao Z, Zhang M, Du Y, Liu J, Zeng G, Li H, et al. Invadopodia in cancer metastasis: dynamics, regulation, and targeted therapies. J Transl Med. 2025;23:548. 10.1186/s12967-025-06526-y. 40380267 10.1186/s12967-025-06526-y PMC12083038

[17] Warnakulasuriya S, Kujan O, Aguirre-Urizar JM, Bagan JV, González-Moles MÁ, Kerr AR, et al. Oral potentially malignant disorders: a consensus report from an international seminar on nomenclature and classification, convened by the WHO Collaborating Centre for Oral Cancer. Oral Dis. 2021;27:1862–80. 10.1111/odi.13704. 33128420 10.1111/odi.13704

[18] Gleber-Netto FO, Yakob M, Li F, Feng Z, Dai J, Kao H-K, et al. Salivary biomarkers for detection of oral squamous cell carcinoma in a Taiwanese population. Clin Cancer Res Off J Am Assoc Cancer Res. 2016;22:3340–7. 10.1158/1078-0432.CCR-15-1761. 10.1158/1078-0432.CCR-15-1761 PMC4930722 26847061

[19] Iocca O, Sollecito TP, Alawi F, Weinstein GS, Newman JG, De Virgilio A, et al. Potentially malignant disorders of the oral cavity and oral dysplasia: a systematic review and meta-analysis of malignant transformation rate by subtype. Head Neck. 2020;42:539–55. 10.1002/hed.26006. 31803979 10.1002/hed.26006

[20] Zhong L, Zhang C, Zheng J, Li J, Chen W, Zhang Z. Increased Cyfra 21 – 1 concentration in saliva from primary oral squamous cell carcinoma patients. Arch Oral Biol. 2007;52:1079–87. 10.1016/j.archoralbio.2007.05.005. 17612501 10.1016/j.archoralbio.2007.05.005

[21] Mahmood H, Shephard A, Hankinson P, Bradburn M, Araujo ALD, Santos-Silva AR, et al. Development and validation of a multivariable model for prediction of malignant transformation and recurrence of oral epithelial dysplasia. Br J Cancer. 2023;129:1599–607. 10.1038/s41416-023-02438-0. 37758836 10.1038/s41416-023-02438-0 PMC10645879

[22] Shephard AJ, Bashir RMS, Mahmood H, Jahanifar M, Minhas F, Raza SEA, et al. A fully automated and explainable algorithm for predicting malignant transformation in oral epithelial dysplasia. Npj Precis Oncol. 2024;8:137. 10.1038/s41698-024-00624-8. 38942998 10.1038/s41698-024-00624-8 PMC11213925

[23] Shaw AK, Garcha V, Shetty V, Vinay V, Bhor K, Ambildhok K, et al. Diagnostic accuracy of salivary biomarkers in detecting early oral squamous cell carcinoma: a systematic review and meta-analysis. Asian Pac J Cancer Prev APJCP. 2022;23:1483–95. 10.31557/APJCP.2022.23.5.1483. 35633529 10.31557/APJCP.2022.23.5.1483 PMC9587865

[24] Khijmatgar S, Yong J, Rübsamen N, Lorusso F, Rai P, Cenzato N, et al. Salivary biomarkers for early detection of oral squamous cell carcinoma (OSCC) and head/neck squamous cell carcinoma (HNSCC): a systematic review and network meta-analysis. Jpn Dent Sci Rev. 2024;60:32–9. 10.1016/j.jdsr.2023.10.003. 38204964 10.1016/j.jdsr.2023.10.003 PMC10776379

[25] Peters MDJ, Marnie C, Tricco AC, Pollock D, Munn Z, Alexander L, et al. Updated methodological guidance for the conduct of scoping reviews. JBI Evid Synth. 2020;18:2119–26. 10.11124/JBIES-20-00167. 33038124 10.11124/JBIES-20-00167

[26] Tricco AC, Lillie E, Zarin W, O’Brien KK, Colquhoun H, Levac D, et al. PRISMA extension for scoping reviews (PRISMA-ScR): checklist and explanation. Ann Intern Med. 2018;169:467–73. 10.7326/M18-0850. 30178033 10.7326/M18-0850

[27] Covidence systematic review software, Veritas Health Innovation, Melbourne, Australia. Available at www.covidence.org.

[28] Landis JR, Koch GG. The measurement of observer agreement for categorical data. Biometrics. 1977;33:159–74. 843571

[29] FDA-NIH Biomarker Working Group. BEST (Biomarkers, EndpointS, and other Tools) Resource. Silver Spring (MD): Food and Drug Administration (US); 2016. 27010052

[30] Hwang JTK, Gu YR, Shen M, Ralhan R, Walfish PG, Pritzker KPH, et al. Individualized five-year risk assessment for oral premalignant lesion progression to cancer. Oral Surg Oral Med Oral Pathol Oral Radiol. 2017;123:374–81. 10.1016/j.oooo.2016.11.004. 28110942 10.1016/j.oooo.2016.11.004

[31] Ali A, Soares AB, Eymael D, Magalhaes M. Expression of invadopodia markers can identify oral lesions with a high risk of malignant transformation. J Pathol Clin Res. 2021;7:61–74. 10.1002/cjp2.182. 33001588 10.1002/cjp2.182 PMC7737762

[32] Jing F, Zhang J, Cai X, Zhou X, Bai J, Zhang H, et al. Screening for biomarkers for progression from oral leukoplakia to oral squamous cell carcinoma and evaluation of diagnostic efficacy by multiple machine learning algorithms. Cancers. 2022;14:5808. 10.3390/cancers14235808. 36497288 10.3390/cancers14235808 PMC9738227

[33] Shams WK, Htike ZZ. Oral Cancer Prediction Using Gene Expression Profiling Mach Learn. 2017;12.

[34] Darling MR, Hwang JTK, Dickson BJ, Cutz J-C, Salama S, McCord C, et al. Assessing oral epithelial dysplasia risk for transformation to cancer: comparison between histologic grading systems versus S100A7 immunohistochemical signature-based grading. Appl Immunohistochem Mol Morphol AIMM. 2023;31:399–405. 10.1097/PAI.0000000000001132. 37249075 10.1097/PAI.0000000000001132

[35] Bashir RMS, Shephard AJ, Mahmood H, Azarmehr N, Raza SEA, Khurram SA, et al. A digital score of peri-epithelial lymphocytic activity predicts malignant transformation in oral epithelial dysplasia. J Pathol. 2023;260:431–42. 10.1002/path.6094. 37294162 10.1002/path.6094 PMC10952946

[36] Ellis BG, Whitley CA, Triantafyllou A, Gunning PJ, Smith CI, Barrett SD, et al. Prediction of malignant transformation in oral epithelial dysplasia using infrared absorbance spectra. PLoS ONE. 2022;17:e0266043. 10.1371/journal.pone.0266043. 35333891 10.1371/journal.pone.0266043 PMC8956195

[37] Liu Y, Li Y, Fu Y, Liu T, Liu X, Zhang X, et al. Quantitative prediction of oral cancer risk in patients with oral leukoplakia. Oncotarget. 2017;8:46057–64. 10.18632/oncotarget.17550. 28545021 10.18632/oncotarget.17550 PMC5542248

[38] Jiang T, Gradus JL, Rosellini AJ. Supervised machine learning: a brief primer. Behav Ther. 2020;51:675–87. 10.1016/j.beth.2020.05.002. 32800297 10.1016/j.beth.2020.05.002 PMC7431677

[39] Zhou Z-H. A brief introduction to weakly supervised learning. Natl Sci Rev. 2018;5:44–53. 10.1093/nsr/nwx106.

[40] Brown LM, Check DP, Devesa SS. Oral cavity and pharynx cancer incidence trends by subsite in the United States: changing gender patterns. J Oncol. 2012;2012:1–10. 10.1155/2012/649498. 10.1155/2012/649498 PMC3345247 22577381

[41] Mello FW, Miguel AFP, Dutra KL, Porporatti AL, Warnakulasuriya S, Guerra ENS, et al. Prevalence of oral potentially malignant disorders: a systematic review and meta-analysis. J Oral Pathol Med. 2018;47:633–40. 10.1111/jop.12726. 29738071 10.1111/jop.12726

[42] Singh MP, Kumar V, Agarwal A, Kumar R, Bhatt MLB, Misra S. Clinico-epidemiological study of oral squamous cell carcinoma: a tertiary care centre study in North India. J Oral Biol Craniofac Res. 2016;6:32–5. 10.1016/j.jobcr.2015.11.002. 10.1016/j.jobcr.2015.11.002 PMC4756063 26937366

[43] Ho P-S, Chen P-L, Warnakulasuriya S, Shieh T-Y, Chen Y-K, Huang I-Y. Malignant transformation of oral potentially malignant disorders in males: a retrospective cohort study. BMC Cancer. 2009;9:260. 10.1186/1471-2407-9-260. 19640311 10.1186/1471-2407-9-260 PMC2734864

[44] la Roi-Teeuw HM, van Royen FS, de Hond A, Zahra A, de Vries S, Bartels R, et al. Don’t be misled: 3 misconceptions about external validation of clinical prediction models. J Clin Epidemiol. 2024;172:111387. 10.1016/j.jclinepi.2024.111387. 38729274 10.1016/j.jclinepi.2024.111387

[45] Moons KGM, Altman DG, Reitsma JB, Ioannidis JPA, Macaskill P, Steyerberg EW, et al. Transparent Reporting of a multivariable prediction model for Individual Prognosis or Diagnosis (TRIPOD): explanation and elaboration. Ann Intern Med. 2015;162:W1–73. 10.7326/M14-0698. 25560730 10.7326/M14-0698

[46] Tejani AS, Klontzas ME, Gatti AA, Mongan JT, Moy L, Park SH, et al. Checklist for Artificial Intelligence in Medical imaging (CLAIM): 2024 update. Radiol Artif Intell. 2024;6:e240300. 10.1148/ryai.240300. 38809149 10.1148/ryai.240300 PMC11304031

[47] Bresnick AR, Weber DJ, Zimmer DB. S100 proteins in cancer. Nat Rev Cancer. 2015;15:96–109. 10.1038/nrc3893. 25614008 10.1038/nrc3893 PMC4369764

[48] Kesting MR, Sudhoff H, Hasler RJ, Nieberler M, Pautke C, Wolff K-D, et al. Psoriasin (S100A7) up-regulation in oral squamous cell carcinoma and its relation to clinicopathologic features. Oral Oncol. 2009;45:731–6. 10.1016/j.oraloncology.2008.11.012. 19147391 10.1016/j.oraloncology.2008.11.012

[49] Nasser MW, Qamri Z, Deol YS, Ravi J, Powell CA, Trikha P, et al. S100A7 enhances mammary tumorigenesis through upregulation of inflammatory pathways. Cancer Res. 2012;72:604–15. 10.1158/0008-5472.CAN-11-0669. 22158945 10.1158/0008-5472.CAN-11-0669 PMC3271140

[50] Zhou G, Xie T-X, Zhao M, Jasser SA, Younes MN, Sano D, et al. Reciprocal negative regulation between S100A7/psoriasin and beta-catenin signaling plays an important role in tumor progression of squamous cell carcinoma of oral cavity. Oncogene. 2008;27:3527–38. 10.1038/sj.onc.1211015. 18223693 10.1038/sj.onc.1211015

[51] Glogauer JE, Sun CX, Bradley G, Magalhaes MAO. Neutrophils increase oral squamous cell carcinoma invasion through an invadopodia-dependent pathway. Cancer Immunol Res. 2015;3:1218–26. 10.1158/2326-6066.CIR-15-0017. 26112922 10.1158/2326-6066.CIR-15-0017

[52] Kato K, Miyazawa H, Kawashiri S, Lambert DW. Tumour: fibroblast interactions promote invadopodia-mediated migration and invasion in oral squamous cell carcinoma. J Oncol. 2022;2022:1–11. 10.1155/2022/5277440. 10.1155/2022/5277440 PMC9719419 36471888

[53] Kaunein N, Ramani RS, Koo K, Moore C, Celentano A, McCullough M, et al. A systematic review of microRNA signatures associated with the progression of leukoplakia with and without epithelial dysplasia. Biomolecules. 2021;11:1879. 10.3390/biom11121879. 34944523 10.3390/biom11121879 PMC8699326

[54] Kojima S, Kuribayashi N, Goda H, Nakashiro K-I, Uchida D. Oral cancer driver gene mutations in oral potentially malignant disorders: clinical significance and diagnostic implications. Discov Oncol. 2025;16:174. 10.1007/s12672-025-01923-7. 39946003 10.1007/s12672-025-01923-7 PMC11825417

[55] The Cancer Genome Atlas Network. Comprehensive genomic characterization of head and neck squamous cell carcinomas. Nature. 2015;517:576–82. 10.1038/nature14129. 25631445 10.1038/nature14129 PMC4311405

[56] Paraskevaidi M, Matthew BJ, Holly BJ, Hugh BJ, Thulya CPV, Loren C, et al. Clinical applications of infrared and Raman spectroscopy in the fields of cancer and infectious diseases. Appl Spectrosc Rev. 2021;56:804–68. 10.1080/05704928.2021.1946076.

[57] Chandu A, Smith ACH, Rogers SN. Health-Related Quality of Life in Oral Cancer: A Review. J Oral Maxillofac Surg. 2006;64:495–502. 10.1016/j.joms.2005.11.028. 16487814 10.1016/j.joms.2005.11.028

[58] Canada H. Guidance Document: Software as a Medical Device (SaMD): Definition and Classification. 2021. https://www.canada.ca/en/health-canada/services/drugs-health-products/medical-devices/application-information/guidance-documents/software-medical-device-guidance-document.html. Accessed 6 Apr 2026.

[59] U.S. Food and Drug Administration. Proposed regulatory framework for modifications to artificial intelligence/machine learning (AI/ML)-based software as a medical device (SaMD): discussion paper and request for feedback. Silver Spring (MD): FDA; 2019.

[60] Chang Y-T, Chu LJ, Liu Y-C, Chen C-J, Wu S-F, Chen C-H, et al. Verification of Saliva Matrix Metalloproteinase-1 as a Strong Diagnostic Marker of Oral Cavity Cancer. Cancers. 2020;12:2273. 10.3390/cancers12082273. 32823758 10.3390/cancers12082273 PMC7463746

[61] Elmahgoub F. Could salivary biomarkers be useful in the early detection of oral cancer and oral potentially malignant disorders, and is there a relationship between these biomarkers and risk factors? Evid Based Dent. 2022;23:30–1. 10.1038/s41432-022-0249-8. 35338326 10.1038/s41432-022-0249-8

