# Supplementary material for: Biomarker-based machine learning for malignant transformation in oral potentially malignant disorders: a scoping review
Source: BMC Oral Health. 2026 Jun 27;26:1381. doi: 10.1186/s12903-026-08943-x (PMC13452107; doi:10.1186/s12903-026-08943-x)
Supplement: Supplementary file 1 [file 12903_2026_8943_MOESM1_ESM.docx]

**Supplementary Materials**

Search Strategies

PubMed:

("Carcinoma, Squamous Cell"[Mesh] OR "Mouth Neoplasms/pathology"[Mesh] OR "oral squamous cell carcinoma" OR "OSCC" ) OR ("Precancerous Conditions"[Mesh] OR "precancerous" OR "OPMD" OR "Oral Potentially Malignant Disorders" OR "Premalignant lesions" OR "Lichen planus oral" OR "Lichenoid eruptions" OR "lichenoid reaction" OR "oral lichenoid mucositis" OR "Leukoplakia oral" OR "Erythroplasia" OR "Erythroplakia" OR "Oral submucous fibrosis" OR "OSMF" OR "Precancerous conditions" OR "Mouth neoplasms" OR "Mouth cancer" OR "Oral Premalignant lesions" OR "Oral Precancerous lesions" OR "Oral Premalignant conditions" OR "Oral Potentially malignant lesions" ) AND ("machine learning" OR "Artificial Intelligence"[Mesh] OR "Automated" OR "artificial intelligence" OR "Straticyte" OR "Deep learning" OR "Random forest algorithm" OR "Computer Neural networks" OR "Prediction tools") AND ("Biomarkers" OR "biomarker").

SCOPUS:

TITLE-ABS-KEY ( ( "oral squamous cell carcinoma" OR "scc" OR "Mouth neoplasms" OR "Mouth cancer" OR "Precancerous Conditions" OR "precancerous" OR "OPMD" OR "Oral Potentially Malignant Disorders" OR "Premalignant lesions" OR "Lichen planus oral" OR "Lichenoid eruptions" OR "lichenoid reaction" OR "oral lichenoid mucositis" OR "Leukoplakia oral" OR "Erythroplasia" OR "Erythroplakia" OR "Oral submucous fibrosis" OR "osm" OR "Precancerous conditions" OR "Oral Premalignant lesions" OR "Oral Precancerous lesions" OR "Oral Premalignant conditions" OR "Oral Potentially malignant lesions" ) AND ( "machine learning" OR "Artificial Intelligence" OR "Automated" OR "artificial intelligence" OR "Deep learning" OR "Random forest algorithm" OR "Computer Neural networks" OR "risk assessment tool" OR "risk assessment tools" OR "risk assessment prediction" ) AND ( "Biomarkers" OR "biomarker" ) )

Web of Science:

TS=(("oral squamous cell carcinoma" OR "OSCC" OR "Mouth neoplasms" OR "Mouth cancer" OR "Precancerous Conditions" OR "precancerous" OR "OPMD" OR "Oral Potentially Malignant Disorders" OR "Premalignant lesions" OR "Lichen planus oral" OR "Lichenoid eruptions" OR "lichenoid reaction" OR "oral lichenoid mucositis" OR "Leukoplakia oral" OR "Erythroplasia" OR "Erythroplakia" OR "Oral submucous fibrosis" OR "OSMF" OR "Precancerous conditions" OR "Oral Premalignant lesions" OR "Oral Precancerous lesions" OR "Oral Premalignant conditions" OR "Oral Potentially malignant lesions") AND ("machine learning" OR "Artificial Intelligence" OR "Automated" OR "artificial intelligence" OR "Deep learning" OR "Random forest algorithm" OR "Computer Neural networks" OR "risk assessment tool" OR "risk assessment tools" OR "risk assessment prediction") AND ("Biomarkers" OR "biomarker"))

Cochrane:

#1 MeSH descriptor: [Carcinoma, Squamous Cell] explode all trees

#2 MeSH descriptor: [Mouth Neoplasms] explode all trees

#3 ("oral squamous cell carcinoma" OR OSCC):ti,ab,kw

#4 (#1 OR #2 OR #3)

#5 MeSH descriptor: [Precancerous Conditions] explode all trees

#6 (precancerous OR OPMD OR "Oral Potentially Malignant Disorders" OR "Premalignant lesions" OR "Lichen planus oral" OR "Lichenoid eruptions" OR "lichenoid reaction" OR "oral lichenoid mucositis" OR "Leukoplakia oral" OR Erythroplasia OR Erythroplakia OR "Oral submucous fibrosis" OR OSMF OR "Precancerous conditions" OR "Mouth neoplasms" OR "Mouth cancer" OR "Oral Premalignant lesions" OR "Oral Precancerous lesions" OR "Oral Premalignant conditions" OR "Oral Potentially malignant lesions"):ti,ab,kw

#7 (#5 OR #6)

#8 MeSH descriptor: [Artificial Intelligence] explode all trees

#9 ("machine learning" OR "artificial intelligence" OR Automated OR Straticyte OR "Deep learning" OR "Random forest algorithm" OR "Computer Neural networks" OR "Prediction tools"):ti,ab,kw

#10 (#8 OR #9)

#11 MeSH descriptor: [Biomarkers] explode all trees

#12 biomarker*:ti,ab,kw

#13 (#11 OR #12)

#14 (#4 OR #7) AND #10 AND #13
